# Facile Control of the Porous Structure of Larch-Derived Mesoporous Carbons via Self-Assembly for Supercapacitors

**DOI:** 10.3390/ma10111330

**Published:** 2017-11-20

**Authors:** Xin Zhao, Wei Li, Honglei Chen, Shoujuan Wang, Fangong Kong, Shouxin Liu

**Affiliations:** 1 Key Lab of Pulp and Paper Science and Technology of Ministry of Education (Shandong Province), Qilu University of Technology, Jinan 250353, China; shaming007@163.com (H.C.); nancy5921@163.com (S.W.); kfgwsj1566@163.com (F.K.); 2 College of Material Science and Engineering, Northeast Forestry University, Harbin 150040, China; liwei19820927@126.com

**Keywords:** larch, mesoporous carbons, soft template, EO/PO ratio, supercapacitors

## Abstract

Mesoporous carbons have been successfully synthesized via self-assembly using larch-based resins as precursors and triblock copolymers as soft templates. The porous structure of mesoporous carbons can be tailored by adjusting the ratio of hydrophilic/hydrophobic (EO/PO) units owing to interfacial curvature. Interestingly, the porous structures show a distinct change from vortex-like to worm-like pores, to stripe-like pores, and to ordered two-dimensional hexagonal pores as the ratio of hydrophilic/hydrophobic units increases, indicating the significant effect of EO/PO ratio on the porous structure. The mesoporous carbons as supercapacitor electrodes exhibit superior electrochemical capacitive performance and a high degree of reversibility after 2000 cycles for supercapacitors due to the well-defined mesoporosity of the carbon materials. Meanwhile, the superior carbon has a high specific capacitance of 107 F·g^−1^ in 6 M KOH at a current density of 10 A·g^−1^.

## 1. Introduction

Due to the increasing sustainable and energy concerns caused by excessive consumption of fossil fuels, energy storage devices, such as fuel cells, lithium-ion batteries, and supercapacitors, are considered alternative candidates for practical applications. Supercapacitors, as promising new-type electrochemical devices with high power density, excellent cyclic performance, and less sustainable development pollution, have attracted considerable attention [1,2,3,4,5]. Generally, supercapacitors can be divided into electrical double-layer capacitors (EDLCs) and pseudocapacitors according to their charge storage mechanism. EDLCs possess an electrostatic attraction at the interface of electrodes and electrolytes with charge accumulation; however, pseudocapacitors exhibit faradic redox reactions [6,7]. The development of supercapacitors is very useful for the large-scale applications of portable electronic systems and automotives due to their high-power density, excellent reversibility, and long cycle life.

Recently, mesoporous carbons have received considerable attention for the development of high-performance supercapacitors due to their high surface area, uniform and tunable porous structure, and chemical inertness [8,9,10]. These unique characteristics make them ideal candidates for supercapacitor electrodes with high power density and energy density. Furthermore, their uniform mesopores facilitate the transportation of electrolyte ions, resulting in better electrochemical performance at high current densities [11,12,13]. Indeed, the mesoporous carbons exhibit significant advantage over the microporous activated carbons under short duration and discharge or recharge [14]. Therefore, the development of ordered mesoporous carbons with high porosity for the supercapacitors is an important issue.

The ordered mesoporous carbons are usually prepared with a hard template method; however, this method is complex, time-consuming, and inefficient, such that it is unsuitable for industrial applications. Recently, the synthesis of ordered mesoporous carbons with the soft template method has attracted increasing attention because it is a facile, time-saving, and efficient strategy. The synthesis technology is based on the self-assembly of copolymer molecules with carbon precursors via hydrogen bond [15,16]. The porous structure of mesoporous carbons prepared with the soft template method can be tuned by the type of solvent, block strength, and carbonization temperature [17,18]. Generally, phenol resins are the main carbon resources for the synthesis of mesoporous carbons via soft template. However, biomasses as renewable resources have limited report for the preparation of mesoporous carbons [19,20,21]. Our group previously reported the synthesis of different morphologies of mesoporous carbons (e.g., carbon films, carbon spheres, and carbon foam) with controllable porous structures using liquefied wood as carbon resources [22,23,24]. Meanwhile, we used triblock copolymer Pluronic F127 and P123 as soft templates for the preparation of the mesoporous carbons with 2-D hexagonal and spherical ordered channel structures via the direct carbonization of organic–organic self-assembled composites [25]. The carbon precursors with abundant hydroxyl groups can strongly interact with the polar parts of structure-directing block-copolymers, leading to the self-assemble of precursors around micelles. These results demonstrated that the wood can be utilized to synthesize the mesoporous carbons with high porosity for supercapacitors.

Herein, we present a facile synthesis of mesoporous carbons with different porous structures (ordered and disordered) via organic–organic self-assembly using liquefied larch as carbon resources and mixture copolymers as templates. The porous structure of the carbons is further tailored by the ratio of hydrophilic/hydrophobic units (EO/PO). Furthermore, we illustrate the mechanism of the self-assembly and the interaction between larch-based resins and templates, in an attempt to attract more attention and determine the real value of these mesoporous carbons. Furthermore, these mesoporous carbons as supercapacitor electrodes are tested to evaluate the capacitive performance, and the effect of the morphology and porous structure of carbons on the electrochemical properties is investigated.

## 2. Experimental

### 2.1. Preparation of Ordered Larch-Based Mesoporous Carbons

The synthesis of the liquefied larch was based on our previous work [25]. In a typical synthesis, larch sawdust (10 g), phenol (30 mL), sulfuric acid (98%, 1 mL), and phosphoric acid (85%, 2 mL) were placed into a three-necked glass. The mixture was heated under reflux at 120 °C for 1 h. Then the mixture was filtered with methanol, and then adjusted to neutral using sodium hydroxide, followed by the filtering of the resulting precipitate. The filtrate was concentrated by vacuum distillation at 40 °C, and liquefied larch was obtained.

The F127 and P123 are expressed with poly(ethylene oxide)-poly(propylene oxide)-poly(ethylene oxide), denoted as EO*_m_*-PO*_n_*-EO*_m_* in which the *m*_1_ and *n*_1_ of F127 is 106 and 70 and the *m*_2_ and *n*_2_ of P123 is 20 and 79, respectively. The EO groups can self-assemble with resins forming mesoporous structure via hydrogen bonds. The EO groups mainly contribute to the pore size of the carbons. For the synthesis of the mesoporous carbons, a mixture of *x* g (0, 2, 4.5, 5.5, 6, 8, and 10 g) F127 and (10 − *x*) g P123 as the soft template was dissolved in 20 mL of ethanol under magnetic stirring at 30 °C. In a typical synthesis, formaldehyde (37%, 90 mL) and sodium hydroxide (3 g) were added to the as-synthesized liquefied larch to generate larch-based resins under basic conditions. Ten-gram templates were then added and stirred at 40 °C for 20 h. Next, the pH was adjusted to 0.5 with HCl, and the reaction was continued at 50 °C for 8 h. The obtained mixture was dried at 80 °C for 6 h. Finally, the carbon materials were formed after carbonization under an *N*_2_ atmosphere at 700 °C for 2 h. The carbon materials were denoted as C − *y*, where *y* is the EO/PO ratio, calculated as follows:

*y* = (*xm*_1_ + (10 − *x*)*m*_2_)/(*xn*_1_ + (10 − *x*)*n*_2_) (1)

where *m*_1_ and *m*_2_ stand for the amount of EO units of F127 and P123, and *n*_1_ and *n*_2_ stand for the amount of PO units of F127 and P123, respectively.

### 2.2. Characterization

The images of transmission electron microscopy (TEM) were obtained on a JEOL 2011 (JEOL, Hokkaido, Japan) apparatus operated at 200 kV. Powder X-ray diffraction patterns of CMs were measured using a Brucker D4 (Bruker, Hokkaido, Japan) powder X-ray diffractometer with Cu Kα radiation at 40 kV and 40 mA. Nitrogen sorption isotherms were measured with a Micromeritics ASAP 2020 sorptometer (Maike, Birmingham, AL, USA) using nitrogen as the adsorbate at 77 K. All samples were degassed at 300 °C for more than 10 h before analysis. The surface area (S_BET_) was calculated using the BET method based on adsorption data in the relative pressure of 0.05–0.2, and total pore volume was determined at the highest relative pressure. The pore size distribution (PSD) was determined via the DFT method using a cylindrical pore model with nitrogen adsorption data.

### 2.3. Electrochemical Measurements

For the fabrication of working electrodes, active materials (80 wt %), carbon black (10 wt %), and polytetrafluoroethylene (PTFE; 10 wt %) were well mixed, which were pressed onto a nickel foam that served as a current collector. The typical mass of active materials was about 10 mg·cm^−2^. The electrochemical experiments were tested in a three-electrode cell, using platinum as the counter electrode, a saturated calomel electrode (SCE) (0.2415 V vs. the standard hydrogen electrode) as the reference electrode, and 6 M KOH solution as the electrolyte. Cyclic voltammetry (CV) measurements and galvanostatic charge/discharge (GCD) tests were carried out on a CHI 600E electrochemical workstation. The CV curves were measured at scan rates of 1–200 mV·s^−1^ in a voltage range of −1 to 0 V. The GCD curves were tested between −1 and 0 V at different current densities. Electrochemical impedance spectroscopy (EIS) measurements were carried out with the amplitude of 5 mV in a frequency range of 10 mHz to 100 kHz.

## 3. Results and Discussion

### 3.1. Structural and Textural Properties of Mesoporous Carbons

**Figure 1:**
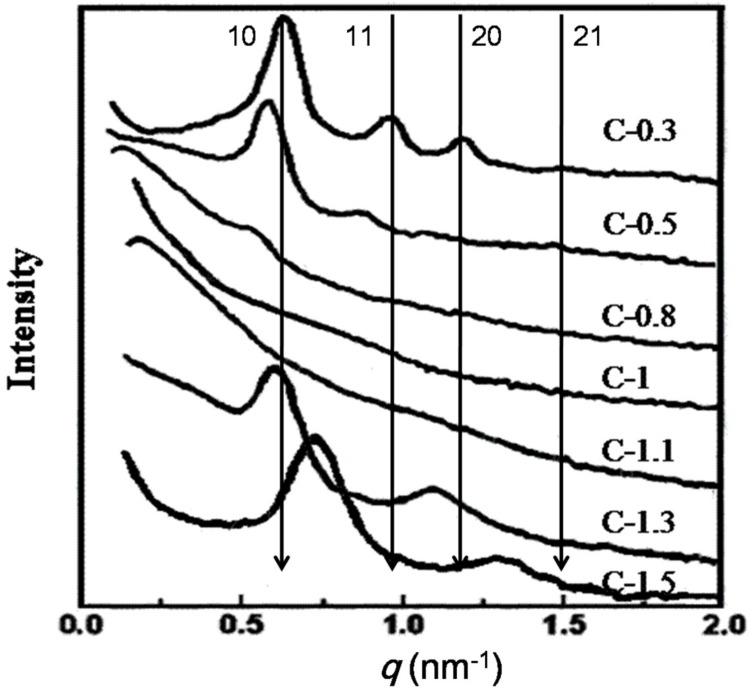
Small-angle X-ray scattering spectra of carbons prepared at different conditions.

Small-angle X-ray scattering (SAXS) patterns of mesoporous carbons are shown in Figure 1. The as-synthesized CM-0.3 shows four scattering peaks with *q* values (*q* = 4πsin*θ*/*λ*) at 0.688, 0.927, 1.208, and 1.493 nm^−1^, which can correspond to the 10, 11, 20, and 21 reflections of two-dimensional (2-D) hexagonal mesostructure (group space, P6m) symmetry. For CM-0.5, the intensity of the four peaks has a slight reduction and shift to a low *q*-value, indicating a decrease in ordering and framework expansion. However, the scattering peaks of CM-0.8 and CM-1 gradually become weak with the increase in the EO/PO, which indicates the change in the ordered porous structure to the disordered structure. Moreover, for CM-1.1, no scattering peak can be observed, indicating a completely disordered structure. Interestingly, the as-obtained CM-1.3 has two evident scattering peaks, indicating the formation of an ordered mesoporous structure. However, the 20 scattering peak shifts to a low *q*-value, indicating framework expansion. Furthermore, the scattering peaks of CM-1.5 gradually become more intensely with the increase in EO/PO, indicating the formation of a highly ordered mesoporous structure. Meantime, the 10 and 20 scattering peaks shift to a high *q*-value, indicating framework shrinkage. These results display the effect of the EO/PO ratio on the formation of porous structures transforming from ordered, to disordered, and then to ordered porous structures.

**Figure 2:**
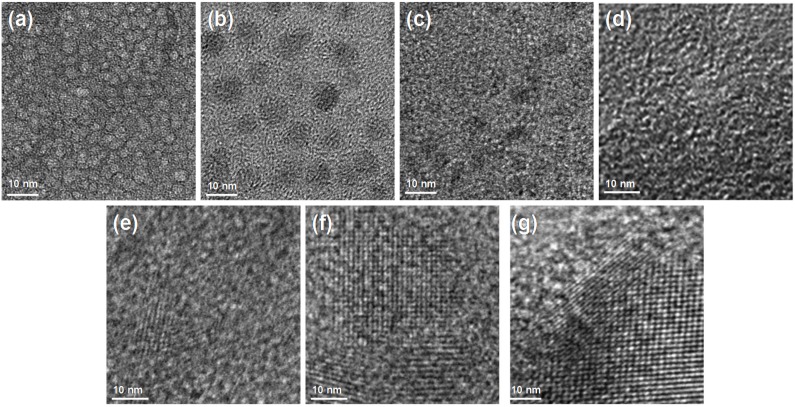
TEM images of carbons prepared at different conditions: (**a**) C-0.3; (**b**) C-0.5; (**c**) C-0.8; (**d**) C-1; (**e**) C-1.1; (**f**) C-1.3; (**g**) C-1.5.

TEM images of mesoporous carbons are showed in Figure 2. It can be seen that the EO/PO ratio has a significant effect on the formation of the mesoporous structures of carbons. The C-0.3 sample possesses vortex-like mesopores, while the amount of vortex-like mesopores reduces and the size of vortex-like mesopores becomes large in the C-0.5 sample. Obviously, with the increase in EO/PO ratio, the porous structure gradually becomes disordered. The C-0.8 sample exhibits a typical disordered mesoporous structure with a few vortex-like pores, and the C-1 sample exhibits a worm-like porous structure. However, ordered porous structures of carbons are gradually formed with the further increase in EO/PO ratio. The stripe-like structure appears when EO/PO reaches 1.1. The C-1.3 sample prepared with EO/PO = 1.3 shows an ordered hexagonal porous structure. Furthermore, a well ordered two-dimensional hexagonal porous structure is formed when EO/PO reaches 1.5, which is consistent with the result of SAXS analysis. The effect of the EO/PO ratio on the formation of porous structures mainly depends on the different interfacial curvature [26]. The formation of well-ordered vortex-like mesopores may be due to the fact that P123 with fewer EO units has high contraction in the axial direction, weakening the interfacial curvature. As the EO/PO ratio increases, the hydrophilic groups gradually increase, leading to the enhancement of interface curvature that can assemble into the ordered stripe-like mesoporous structure. The weaker interfacial curvature prefers to aggregate into vortex-like microdomains, while the higher interfacial curvature prefers to aggregate into stripe-like microdomains [27].

**Figure 3:**
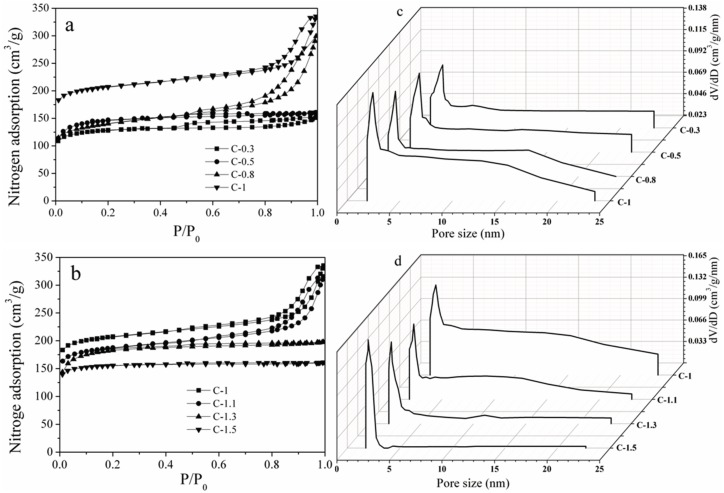
Nitrogen sorption isotherms (**a**,**b**) and pore size distribution curves (**c**,**d**) of carbons prepared at different conditions.

The *N*_2_ sorption isotherms and the corresponding pore size distribution curves of mesoporous carbons are displayed in Figure 3. The all samples exhibit a significant *N*_2_ adsorption at the relative pressure (*P*/*P*_0_) below 0.1, indicating the formation of abundant micropores. As shown in Figure 3a, the amount of the *N*_2_ adsorption at *P*/*P*_0_ = 0.1 gradually become larger with the increase in EO/PO ratio from 0.3 to 1. Meanwhile, a hysteresis loop in a *P*/*P*_0_ range of 0.45–1.0 is observed, indicating a typical characteristic of mesoporous structure. The hysteresis loop of C-1 can be categorized into the H_3_-type isotherm according to the IUPAC classification, which indicates that the C-1 sample possesses slit pores and some large pores. The C-0.8 sample shows a slight change in the hysteresis loop and a distinct decrease in nitrogen sorption at a relative pressure *P*/*P*_0_ below 0.1, indicating the main decrease in micropores. However, the hysteresis loops of C-0.5 and C-0.3 have a distinguishing change, which can be classified as an H_1_-type isotherm, indicating the formation of vortex-like pores with a narrow pore size distribution. Furthermore, the H_1_-type hysteresis loop of the C-0.3 sample increases in the *P*/*P*_0_ range of 0.42–1.0, and a slight decrease in nitrogen sorption at a relative pressure *P*/*P*_0_ below 0.1 shows the increase in mesopores. Compared with the C-1 sample (Figure 3b), the C-1.1 sample shows a slightly decrease in nitrogen sorption at a relative pressure *P*/*P*_0_ of 0.1–0.45, and the H_1_-type hysteresis loop slightly changes. However, the H_3_-type hysteresis loop of the C-1.3 sample distinctly reduces, indicating the decrease in mesopores. Moreover, the C-1.5 sample exhibits a small H_1_-type hysteresis loop, indicating the formation of mesoporous carbons. These results demonstrate that the EO/PO ratio is the key issue on the formation of porous structure. The carbons prepared with EO/PO = 1 shows the highest adsorption. This is probably due to the synergistic effect of copolymers forming abundant porous structures. The mixture of EO and PO blocks can affect the formation of interconnected microtunnels between the larch resins and the copolymers [28]. The pore size distribution curves of carbons are shown in Figure 3c,d. The C-0.3 sample possesses the maxima centered at 1.2, 3.5, and 6.4 nm. The pores of C-1 sample become wider and centered at 1.3, 3.4, and 17.2 nm with the broadest pore size distribution. This is because of the collapse of pores caused by the synergistic effect of EO and PO groups after carbonization. The maxima of carbons prepared with high EO/PO ratio also gradually shifts to a narrow pore, and C-1.5 has maxima centered at 1.2 and 3.4 nm, which is relative to the amount of PO groups. These results indicate that the EO/PO ratio has a significant effect on the formation of porous structures.

The textual parameters of the C-y materials are presented in Table 1. The C-1 sample possesses the highest S_BET_ (up to 634 m^2^·g^−1^), and the S_meso_/S_BET_ of C-1 reach 20%. This is due to the strong synergistic effect of equaled EO and PO units. As the EO/PO ratio decreases from 1 to 0.3, the S_BET_ of carbons decreases from 637 to 398 m^2^·g^−1^, and the S_meso_/S_BET_ ratio decreases from 20% to 14%. The S_BET_ of carbons decreases from 637 to 475 m^2^·g^−1^, and the S_meso_/S_BET_ decreases from 20% to 12% when the EO/PO increases from 1 to 1.5, respectively. These results indicate that the EO/PO ratio is of great importance in controlling the porous structure.

### 3.2. Formation Mechanism of Mesoporous Carbons

**Figure 4:**
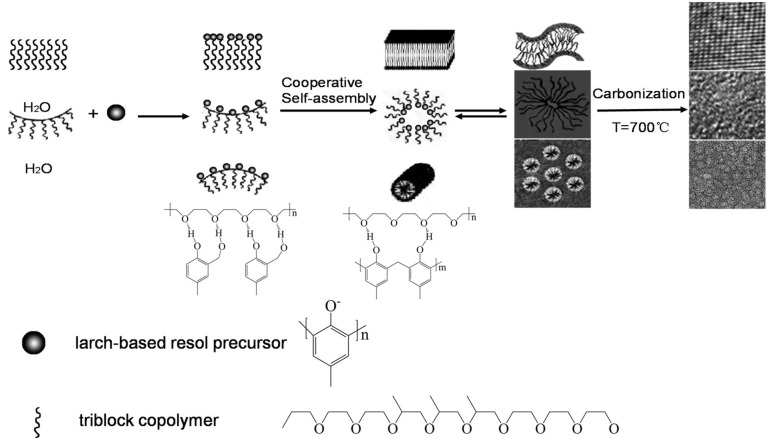
Schematic illustration of the proposed mechanism for the formation of the mesoporous carbons.

Mesoporous carbons with different porous structures have been synthesized via a facile method, and the formation of the mesostructure is related to the co-operative self-assemble mechanism, as shown in Figure 4. We prepared the liquefied larch via phenol liquefication, which has a condensation reaction with formaldehyde to form larch-based resins under basic conditions. The obtained resin precursors with abundant hydroxyl can further interact with EO units via hydrogen bond forming resin-copolymers composites, and the copolymer micelles penetrate into the EO–PO interface, which assembles in large voids to form mesopores with various ordered mesostructure symmetries [29]. After carbonization at 700 °C, soft templates are removed and the corresponding mesopores are opened. Additionally, the hydrophilic and hydrophobic segments have a strong interaction with each other, which can affect the porous structure of carbons. When the EO units are equal to the PO units, the composites have a strong interaction due to their synergistic effect [30], resulting in the collapse of the mesostructure after carbonization. The stripe-like micelles are formed when the ratio of EO/PO is bigger than 1, which is due to the strengthening ability of the hydrophilic segment with high interface curvature. The high interface curvature can maintain the stability of the stripe-like micelles and form a rigid framework after carbonization. On the other hand, the vortex-like micelles are formed when the ratio of EO/PO is smaller than 1, which is ascribed to the strengthening ability of the hydrophobic segment surrounding the hydrophilic segments with low interface curvature. The low interface curvature can maintain the stable spherical micelles and form rigid framework after carbonization.

### 3.3. Electrochemical Properties

**Figure 5:**
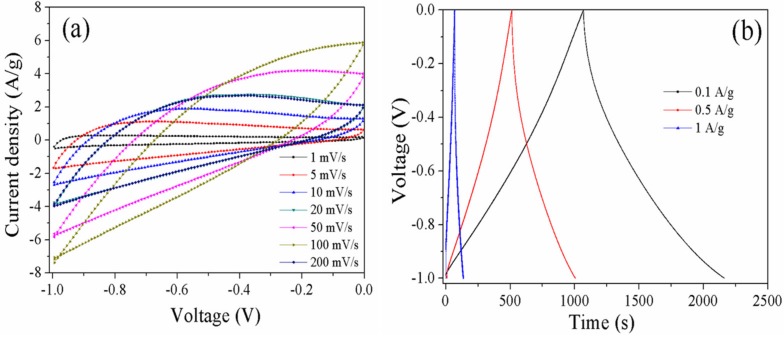
CV curves of C-0.3 at different scan rates varying from 1 to 200 mV·s^−1^ (**a**) and charge–discharge curves at different current densities from 0.1 to 1 A·g^−1^ (**b**).

The mesoporous carbons with well-developed porosity were also evaluated for electrode materials in supercapacitors. The cyclic voltammetry and galvanostatic charge/discharge tests were employed to characterize the capacitive properties. Figure 5a shows the cyclic voltammetry curves of C-0.3 at scan rates from 1 to 200 mV·s^−1^. The cyclic voltammetry curves measured at low scan rates show a nearly rectangular shape, suggesting a double-layer capacitance behavior. However, the shape significantly changes from a rectangular shape to polar curves with the increase in scan rate, indicating the low conductivity of carbons. The charge–discharge plots of C-0.3 measured at current densities from 0.1 to 1 A·g^−1^ are shown in Figure 5b. The charge–discharge curves have a distinct arc with a small IR drop due to the low conductivity of the electrode materials. The specific capacitance of C-0.3 is calculated using discharge plots, which is 79 F·g^−1^ at a current density of 1 A·g^−1^. This indicates that C-0.3 possesses a poor capacitive performance.

**Figure 6:**
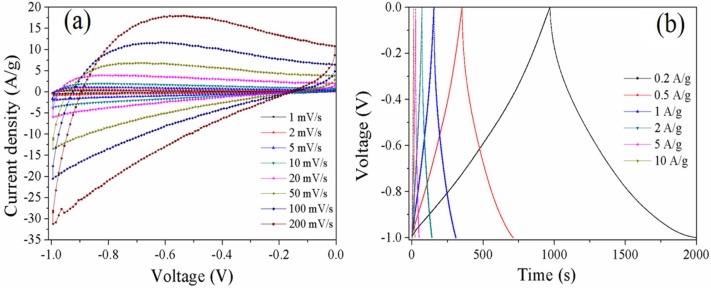
CV curves of C-1 at different scan rates varying from 1 to 200 mV·s^−1^ (**a**) and charge–discharge curves at different current densities from 0.2 to 10 A·g^−1^ (**b**).

As can be seen in Figure 6a, the CV curves of C-1 show a nearly rectangular shape at the low scan rates, suggesting a double-layer capacitance behavior. However, the shape slightly changes from a rectangular shape to a polar curve with the increase in scan rate, indicating the low conductivity of carbons. The charge–discharge plots of C-1 measured at current densities from 0.2 to 10 A·g^−1^ are shown in Figure 6b. The charge–discharge curves have a distinct arc with a small IR drop due to the low electronic conductivity of the electrode materials. The specific capacitance of C-1 measured at a current density of 1 A·g^−1^ is 158 F·g^−1^, which is better than that of C-0.3. However, the specific capacitance only retains 103 F·g^−1^ when the current density increases to 10 A·g^−1^. This indicates that C-1 possess a moderate capacitive performance and poor electronic conductivity.

**Figure 7:**
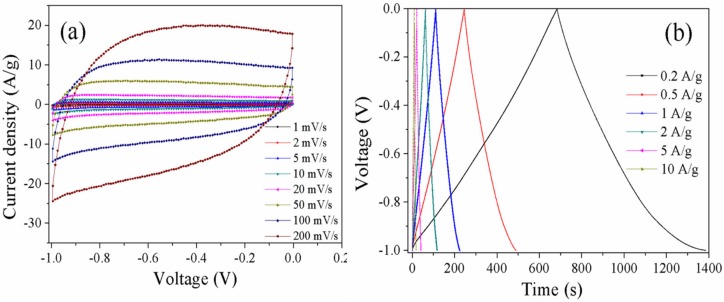
CV curves of C-1.5 at different scan rates varying from 1 to 200 mV·s^−1^ (**a**) and charge–discharge curves at different current densities from 0.2 to 10 A·g^−1^ (**b**).

The CV curves of C-1.5 showed in Figure 7a possess a nearly rectangular shape in the low scan rates, indicating a double-layer capacitance behavior. Furthermore, the shape has no apparent change with the increase in scan rate, indicating excellent conductivity due to the ordered mesoporous structure. The charge–discharge plots of C-1.5 measured at current densities from 0.2 to 10 A·g^−1^ are shown in Figure 7b. The charge–discharge curves show isosceles triangles due to the excellent conductivity of the electrode materials. The specific capacitance of C-1.5 measured at a current density of 1 A·g^−1^ is 125 F·g^−1^, and the specific capacitance retains 107 F·g^−1^ when the current density increases to 10 A·g^−1^, indicating superior conductivity due to the well-defined porosity. The capacitance drop of all the carbons at high current density can be illustrated by the assumption that the charge diffusion in the pores is interrupted owing to the time prevent to rate of charge/discharge [31].

**Figure 8:**
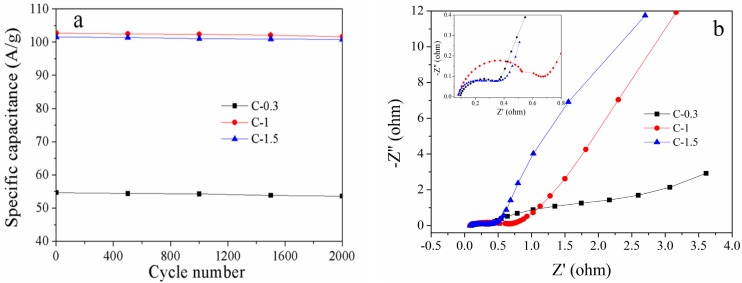
The cycling stability measured at 10 A·g^−1^ (**a**) and Nyquist plots of impedance spectra (**b**) of the mesoporous carbons.

To further investigate the electrochemical performance of the carbons, the circulations and EIS were systematically evaluated. The cycling stability tests of the carbons were performed at a current density of 10 A·g^−1^ for 2000 galvanostatic charge/discharge cycles, as shown in Figure 8a. It can be seen that the specific capacitances of the carbons decrease slightly and remain above 98% of the maximum capacitance after 2000 cycles, indicating that the electrode has good electrochemical stability and a high degree of reversibility due to the presence of the meropores. Electrochemical impedance spectroscopy (EIS) analysis was used to gain a deep insight into the resistive and capacitive behavior of the carbons. As shown in Figure 8b, the slope of the curves shows an electric double-layer capacitive behavior at low frequencies. However, C-1.5 has the highest slope, indicating the lowest resistance. In the region of medium frequencies, the Warburg impedance of all samples is observed, where the electrolyte ion penetrate into the depth of the micro-/mesopores of the electrode [32]. At high frequencies, the capacitive behavior in EIS is illustrated by the semi-circle plots, a small semicircle indicates a low contact resistance, which is related to the interaction between porous structure of the electrode and ions [33]. This is related to the interaction of the porous structure of the electrode and ions. C-0.3 and C-1.5, compared to C-1, have smaller radii due to the presence of order mesopores. At high frequencies, the capacitive performance is poor because it is difficult to access the rapid variation of the potential by the transfer of abundant ions [34]. In the region of medium frequency, the Warburg impedance of samples were observed, where the electrolyte ions penetrate into the depth of the pores of the electrode. At a low frequency, a straight line demonstrates the ideal electric double-layer capacitive behavior of electrode materials [35]. The low internal resistance has a positive effect on conductivity. These results correspond to the analysis of CV and GCD curves. Consequently, C-1.5 has well-developed porosity and a high surface area, resulting in low resistance, which exhibits excellent electrochemical performance and shows potential for industrial applications.

## 4. Conclusions

We report a facile and reproducible approach for the synthesis of mesoporous carbons with different porous structures and tunable pore sizes by using a mixture of F127 and P123 as templates and larch-based resins as carbon sources. The change in porous structure from worm-like to 2D hexagonal structure was observed with the increase in EO/PO ratio from 1 to 1.5. However, the porous structure changes from a worm-like to a vortex-like porous structure when EO/PO ratio decreases from 1 to 0.3. The mesoporous carbons where EO/PO = 1 have the highest surface area. Meanwhile, the pore size of all carbons is mainly distributed at 1.8–3.0 nm. The S_BET_ of carbons decreases from 634 to 398 m^2^·g^−1^ as the EO/PO ratio decreases from 1 to 0.3. The S_BET_ of carbons decreases from 634 to 475 m^2^·g^−1^ when the EO/PO increases from 1 to 1.5. Indeed, the pore size depends on the length of the hydrophobic PO blocks in the mixture of F127 and P123. The probable cooperative assembly mechanism of mesoporous carbons is proposed for the synthesis route. Furthermore, the electrochemical experimental results demonstrate that the mesoporous carbons exhibit high specific capacitance, superior electrochemical stability, and a high degree of reversibility. In addition, the carbon where EO/PO = 1.5 has a high specific capacitance of 107 F·g^−1^ in 6 M KOH at a current density of 10 A·g^−1^, and the carbon where EO/PO = 1.5 exhibits excellent conductivity, which is suitable for industrial applications.

## Figures and Tables

**Table 1:** Textual parameters of carbons prepared at different conditions.

Sample	S_BET_ (m^2^/g)	S_meso_/S_BET_ (%)	S_micro_/S_BET_ (%)
C-0.3	393	14	86
C-0.5	410	17	83
C-0.8	421	20	80
C-1	634	20	80
C-1.1	601	18	82
C-1.3	569	14	86
C-1.5	475	12	88
